# Research on the influencing factors of user engagement in tourism short video from the perspective of configuration

**DOI:** 10.1371/journal.pone.0337406

**Published:** 2025-12-11

**Authors:** Lingyan Li, Haoyu An

**Affiliations:** School of Economics and Management, Yanshan University, Qinhuangdao Hebei, China; Shandong Jiaotong University, CHINA

## Abstract

Tourism short videos have become a crucial means for disseminating information and promoting tourism destinations, with user engagement directly impacting their spread and the long-term development of tourism industries. Taking Xi’an as a case study, this paper constructs a framework for influencing factors of user engagement in tourism short videos from three dimensions: content, society, and technology, based on the theory of communication ecology. By blending multiple regression analysis with fuzzy-set qualitative comparative analysis (fsQCA), we explore the factors that influence user engagement in tourism short videos and further clarify the configurational causal relationships among these factors. This study found that: Content level (video type, scene quantity, scene representation, title sentence), social level (blogger type, blogger influence, blogger activity), and technological level (narrative way, background music, advertising information) significantly impact user engagement in tourism short videos. No single factor can solely constitute a necessary condition for promoting high user engagement. User engagement is actually the result of a combination of multiple factors. Notably, social level factors play a pervasive role in fostering active user engagement in tourism short videos. Further, fsQCA analysis identifies six conditional configurations that lead to high user engagement, categorizing them into three path types: content-driven, dual-dimensional co-driven, and comprehensive linkage. This study reveals the complex logic behind user engagement in tourism short videos, providing valuable insights for content marketing and promotional strategies of tourism short videos.

## Introduction

In recent years, short videos have experienced explosive growth, swiftly integrating into and permeating the daily lives of the masses. Short videos have become a highly popular form of leisure and entertainment, deeply influencing the allocation and reconstruction of people’s time and living scenarios. The tourism industry has gradually changed its traditional content production methods, embracing short videos for tourism promotion and marketing. Users have demonstrated immense interest in tourism short video content. According to the “2023 TikTok tourism industry white paper”, the scale of users interested in tourism on short video platforms has exceeded 400 million, and user interaction metrics related to tourism content are also showing an upward trend. Leveraging the powerful content dissemination carrier of tourism short videos, destinations can maximize their charm through online platforms, rapidly expanding their popularity and influence, thereby attracting more users’ attention and visits. However, with the surge in tourism short videos, several accompanying issues have gradually emerged. Destination short video marketing faces challenges such as aesthetic fatigue from promotional routines, difficulty in precisely reaching potential users, and low marketing conversion rates. As tourism marketing enters a stage of high-quality development, it is essential to grasp core attractions, satisfy user demand positioning, and evoke users’ interests and emotional perceptions. By doing so, tourism short videos can stimulate widespread user engagement and social interaction, effectively leveraging the marketing effectiveness of tourism short videos. Therefore, to promote the sustainable development of destination tourism short videos, it is urgent to deeply insight into and grasp users’ real expectations and preferences for tourism short videos. By aligning with tourist demand, tourism short videos can maximize their effectiveness in destination marketing and promotional strategies.

The openness of tourism short video platforms provides users with opportunities to express opinions, disseminate information, and engage in online interactions [1]. Due to the ubiquitous presence of information asymmetry, individuals often rely on their perceptual systems as the primary channel for information exchange and behavioral responses. According to the theory of tourism marketing perception, tourists form specific dynamic evaluations of tourism marketing information stimuli based on their own perceptual experiences, and subsequently make corresponding behavioral choices. In the specific media form of tourism short videos, destination marketers utilize short video marketing techniques to convey a wealth of tourism product and service information to tourists. When audiences browse these tourism short videos, they form an overall cognition and impression of the destination’s tourism content through sensory contact, information reception, and interactive experiences. This multidimensional perception can influence people’s behavioral manifestations. People browse and watch tourism short videos online, like and collect interesting content, and participate in discussions and sharing of popular topics [2]. Behind these engagement behaviors lie users’ interests, attitudes, and preferences, from which a vast amount of potential information can be captured [3]. Analyzing user engagement behaviors in tourism short videos can deeply explore the real needs of tourists, which is of great value for guiding targeted marketing of destinations [4]. However, discussions on user engagement in the tourism field mostly focus on platforms such as tourism websites and online communities, with insufficient exploration of the intrinsic mechanisms of user engagement behavior in tourism short videos which is an important medium for cultural tourism promotion. Although some scholars have explored the driving factors of tourism short videos user engagement from different perspectives, a comprehensive and integrated perspective for in-depth and systematic analysis of the influencing mechanisms of destination tourism short video user engagement behaviors is lacking.

The theory of communication ecology views the information dissemination process as a complex and dynamic ecosystem composed of three interrelated elements: content, society, and technology [5]. Destination tourism short videos, as audiovisual products generated by the fusion of multimodal elements, possess multiple characteristics, including content attributes, relational attributes, and technical attributes. The comprehensiveness and integration of communication ecology theory can provide an overall perspective for exploring the influencing factors of user engagement in tourism short videos. Therefore, this paper will apply this theory to explore user engagement behaviors in destination tourism short videos from an integrated perspective of content, society, and technology. Additionally, the complexity and diversity of users’ psychological activities leads to corresponding complexity in tourism short video user engagement behaviors. Existing studies mostly conduct unilateral analyses of user engagement in tourism short videos, with less consideration of the internal connections between various elements. It is currently unclear whether a single factor can independently influence user engagement in tourism short videos, whether there exist combinations of factors that effectively promote users’ active engagement, and the multifactorial paths that form high user engagement in destination tourism short videos have not been revealed. Therefore, it is urgent to clarify the interactive influence mechanisms among the factors affecting user engagement in destination tourism short videos. It is necessary to adopt the fuzzy-set qualitative comparative analysis(fsQCA) method to deeply explore the interactions between various factors affecting user engagement in short videos and investigate the key paths that can stimulate high user engagement behaviors.

In summary, this study selects Xi’an, a popular travel destination ranking in the top ten nationwide in terms of online check-ins and views, as a case study. Based on the theories of tourism marketing perception, communication ecology, and user engagement behavior, combined with the constitutive characteristics of short video, this research comprehensively explores the influencing factors of user engagement from content, social, and technological dimensions. The paper establishes a research framework for the influencing factors of user engagement in destination tourism short videos. From a configurational perspective, this study employs the fsQCA method to investigate the pathways that lead to active user engagement, clarifying the complex mechanisms underlying the multiple concurrent causes of user engagement behavior. By accurately grasping users’ psychology and needs, this study provides insights for destinations to formulate more targeted short video marketing strategies, thereby enhancing their attractiveness and influence and promoting high-quality development of the tourism industry.

### Literature review

In the era of the digital economy, short videos have revolutionized the traditional content production methods of the tourism industry, becoming an essential channel for promoting tourist destinations. Their powerful traffic-drawing effect gave rise to a multitude of online famous landmarks. Compared to graphic and text content, short videos are more straightforward and direct in stimulating the sensory experiences of the audience. Browsing tourism short videos has become a primary way for the public to obtain tourism information and to make travel decisions [6]. Users can gain intuitive, concrete, and diversified understandings of tourist destinations through social media platforms [7]. Online interactivity has become a key aspect for the public to perceive destination images, exchange travel experiences, and accumulate travel information. User engagement is a process where users take initiative [8], and in the context of tourism short videos, it is viewed as online interaction on social media, manifested through likes, comments, collects, and shares as feedback on the videos [9]. These active interactive engagement behaviors complement passive engagement behaviors like viewing and browsing [10], reflecting the external projection of users’ internal preferences for tourism short videos. However, current research mainly focuses on traditional modalities such as tourism texts and images, with relatively inadequate exploration of tourism videos, especially regarding the user engagement mechanism for destination tourism short videos. User engagement behavior not only affects the dissemination of tourism short video content on social media platforms [11] but also relates to the marketing effectiveness of tourist destinations. Therefore, discussing users’ engagement behavior in tourism short videos has become very important.

The multidimensional perception of tourism short video marketing by individuals is a crucial condition for driving their engagement behavior. Regarding the factors influencing user engagement behavior, existing research primarily focuses on the perspectives of the information itself, such as video production style [12] and content effectiveness [13], external environmental factors like short travel video bloggers [14] and the informational environment, as well as individual internal factors including users’ contextual conditions of use [15] and personal motivations [16]. However, user engagement in tourism short videos is influenced by various aspects. Previous studies have been conducted from specific or limited aspects, lacking a multidimensional and integrated perspective that encompasses different levels. The communication ecology theory (CET), first proposed by Altheide, suggests that the ecology of communication is an organic unity of information technology, communication paradigms, and social behaviors rather than just the effects of single elements. This theory places the information dissemination process within the social ecological system, emphasizing the interactive relationships among the various environments involved in information dissemination. Considering the roles of different individuals, media, and social structures in modern media dissemination, Foth and Hearn proposed three levels of communication ecology [5,17]: content level, social level, and technical level, exploring issues in the field of information systems from a more holistic perspective. Among these, the content level pertains to the themes and actual content of communication, the technological level consists of communication media and technical equipment, and the social level involves the social relationships among different individuals and groups involved in communication. Tourism short videos are not limited to the single attribute of traditional information processing. Their functions have extended to areas such as dissemination, interaction, and consumption. The comprehensiveness of the communication ecology theory provides a clear and holistic framework for studying user engagement behavior in tourism short videos. Therefore, it is necessary to introduce the communication ecology theory as an analytical framework to explore the factors influencing user engagement behavior of destination tourism short videos from more complete and diverse dimensions such as content, social, and technical levels.

Most existing studies have utilized methods such as surveys [14] and interviews [16] to analyze user engagement. However, these methods are often influenced by the subjectivity of respondents and have limited sample sizes, making it difficult to capture the feature of user behavior. The development of big data provides a vast data source and a more comprehensive user profile, facilitating deeper insights into users’ underlying psychological needs and behavioral preferences. Furthermore, user engagement behavior in destination tourism short videos is driven by multiple factors. Traditional research methods struggle to comprehensively interpret the reasons for user engagement in short videos and their interrelations, necessitating a more comprehensive analytical framework and approach to dissect complex user engagement behaviors. Miller first proposed the concept of configuration, arguing that any outcome is the result of complementary and interactive elements, with each element acting within the overall configuration rather than in isolation [18]. Configuration theory shifts from a one-dimensional perspective to a systemic one, no longer confined to examining the “net effect” of a single variable on a dependent variable. Instead, it focuses on exploring the complex relationships between causes and effects. This multivariable causality within systems is characterized by asymmetry, meaning that different antecedent conditions can combine in various ways to produce the same or different outcomes. With the rise of the configuration theory, qualitative comparative analysis (QCA) emerged as a method to explore the configurational effects of multiple concurrent causes through set relations, effectively addressing and explaining the complexity of causal relationships [19]. It has demonstrated advantages not only in studies with small to medium sample but also in large samples cases. Given the advantages of configuration theory and QCA method in addressing complex causal issues, they can compensate for the inadequacies of single-factor explanations of the complex motivations behind user engagement behavior. Therefore, it is necessary to investigate the complex causal mechanisms of user engagement in destination tourism short videos from a configurational perspective. Fuzzy-set qualitative comparative analysis (fsQCA) is a type of QCA method that treats variables as fuzzy membership values between 0 and 1. It leverages set theory and boolean algebra operations to reveal the impact of antecedent conditions on outcomes. It can handle both continuous and categorical variables simultaneously and allows for the existence of partial membership relations. Based on the research questions and variable types in this study, fsQCA is chosen to investigate how various factors interact to influence user engagement behavior in Xi’an tourism short videos.

In summary, existing research has primarily focused on tourist behavior in traditional modalities, with a notable lack of studies examining user engagement with destination tourism short videos. The dissemination of tourism short videos is a complex process, involving diverse perceptions by tourists, and there is a dearth of integrated analyses that encompass content, social, and technological aspects. Furthermore, current research predominantly investigates the correspondence between independent and dependent variables, making it difficult to fully decipher the complex causal relationships underlying user engagement with destination tourism short videos when independent variables interact. Therefore, this study takes Xi’an, one of the top ten influencer cities with the highest popularity on short video platforms, as an example. By integrating computer vision analysis technology into the process of mining tourist behavior preferences in destination tourism short videos, and fully considering the complexity of user engagement with tourism short videos, this study constructs a research framework for user engagement behavior in destination tourism short videos from the perspective of tourist perception, in conjunction with communication ecology theory. Utilizing a mixed-method approach, this study systematically explores the influencing factors of user engagement, uncovers the configuration paths that stimulate user engagement in destination tourism short videos, and thereby assists destination marketers in understanding user needs and preferences to achieve more effective tourism short video promotion and marketing.

### Research design

#### Research area.

Driven by mobile short video platforms, cities of varying scales and development speeds are being rediscovered in diverse forms by the public, shaping a variety of fresh and unique urban labels. Leveraging its historical background and cultural icons, Xi’an has emerged as one of the most popular cities through tourism short videos, sparking widespread online attention and offline visits. Topics related to Xi’an cuisine and tourism have garnered hundreds of billions of views on short video platforms, with multiple attractions featured on TikTok’s popular check-in destinations. Tourism short videos have become a crucial medium for Xi’an’s external promotion, with both official media and the general public playing key roles in showcasing the city’s charm through these videos. Making short videos more attractive and identifying the patterns that drive user engagement in tourism short videos are crucial for promoting high-quality development of Xi’an’s tourism economy. Therefore, this paper takes Xi’an’s tourism short videos as an example to identify factors influencing user engagement and to explore which combinations of factors can elicit higher levels of user engagement.

### Construction of research framework

In the context of tourism short videos, user engagement manifests as multifaceted input and responses by users on short video platforms [20]. Based on differences in behavioral attitudes and initiation modes, user engagement can be divided into continuous browsing and active engagement. Continuous browsing is largely a passive process where users follow the recommendation mechanisms of the platform, whereas active engagement is a positively spontaneous behavior, often manifesting through a series of actions such as liking, commenting, collecting, and sharing [15]. Therefore, this study focuses on users’ active engagement behaviors, comprehensively considering indicators such as likes, comments, collects, and shares to objectively measure user engagement in destination tourism short videos. The overall level of user engagement in Xi’an tourism short videos is quantified by calculating user engagement scores.

As an information dissemination medium, the core of destination tourism short videos lies in outputting content in video format to convey rich tourism information dynamics. Moreover, the essence of short videos is a model of shared and co-created content by all, with its inherent social characteristics breaking the isolation between individuals and enabling various entities to form an open and closely connected network. Additionally, technological development is a significant force driving the vigorous development of short videos, and the fusion of multiple technologies offers more possibilities, creating many unprecedented new scenarios. It can be seen that user engagement with destination tourism short videos is easily influenced by factors such as video content, social relationships, and platform technology, which correspond to the three levels in communication ecology theory. Specifically, the content level of tourism short videos primarily focuses on the basic attribute characteristics and core content presentation conveyed by the videos. The social level mainly involves the key information and creative performance of content creators who are fundamentally connected to the user groups in the social media chain. The technical level encompasses various technical means and forms of expression involved in the production and dissemination of short videos. Therefore, based on communication ecology theory and the components of short videos, this paper explores factors that may stimulate user engagement in destination tourism short videos from the content, social, and technical levels, and constructs a theoretical framework of influencing factors for user engagement behaviors in destination tourism short videos.

The content level refers to the content characteristics of destination tourism short videos, which are crucial to the success of information dissemination. It primarily involves the content elements that are directly presented to users when they browse short videos, including titles, timestamps, video frames, and durations. Firstly, the theme type of the short video is its core, and only valuable and in-demand topics can gain wider user recognition. Meanwhile, the scenes of tourism short videos refers to the visual presentation constructed through the combination of subjects, environments, and spaces, showcasing specific tourism elements of the destination. With the development of the mobile internet, the content presentation of short video has become increasingly diverse, with the clever embedding of various distinctive scenes that leave a deeper impression of the destination on the audience. The number of short video scenes and their representational content determines the user’s experience and influences their engagement behaviors. Secondly, the title is the first impression for users, and its sentence structure carries distinctive emotion, which varies in their attractiveness to users. The length of the title also relates to users’ ability to capture information. Short titles can present key information quickly, while long titles can convey more detailed content [21]. Furthermore, a short video’s duration can range from a few seconds to several minutes, and the length may affect attracting and maintaining users’ attention. In summary, based on existing research and the presentation characteristics of short video browsing interfaces, this study selects variables at the content level including video type, scene quantity, scene representation, title sentence, title length, and video duration.

The social level is related to people and social structures in the information dissemination process. User engagement behavior in tourism short videos is often influenced by multiple dimensional attributes of the creator’s account type, influence, and activity level. Among these, the type of blogger is associated with their content production mode. Different types of short video creators can elicit different reactions and engagement levels from users. Officially-generated content(OGC) typically carries higher authority, professionally-generated content(PGC) combines professionalism with entertainment, while user-generated content(UGC) tends to be more authentic and reliable. The influence of bloggers is closely related to their follower count, which not only determines the dissemination scope of short videos but also reflects their popularity among users. Blogger activity level is measured by the number of works they produce, and maintaining an appropriate frequency of short video postings is essential to retain good exposure and user stickiness. Therefore, this paper selects blogger type, blogger influence, and blogger activity as research variables at the social level.

The technical level is closely related to the diverse functionalities provided by platforms. As a composite media format, tourism short videos rely on technical support from short video platforms for shooting, editing, and production, resulting from the combined effects of various additional functionalities and multimodal symbols such as language, screen, and music. Firstly, the narrative of short videos is an important supplement to video information, enabling a more complete and rich expression of the video content. Different narrative ways can affect the quality of user information acquisition and understanding, thereby influencing user engagement with the short videos. Secondly, audio elements are crucial to user experience, and background music can enrich the audiovisual effects of the short video. Traditionally, short videos exist in both horizontal and vertical screen formats. The vertical format aligns with the natural screen ratio of mobile phones, effectively focusing the image and enlarging details, making it more suitable for users’ habits. Additionally, as online consumption continues to develop, advertising and shopping functionalities on short video platforms have become important channels for the online sales of tourism products. Some creators attach group-buying advertisements when posting short videos, which may influence user engagement [22]. Therefore, this paper focuses on four variables at the technical level: narrative way, background music, video format, and advertising information.

**Fig 1:**
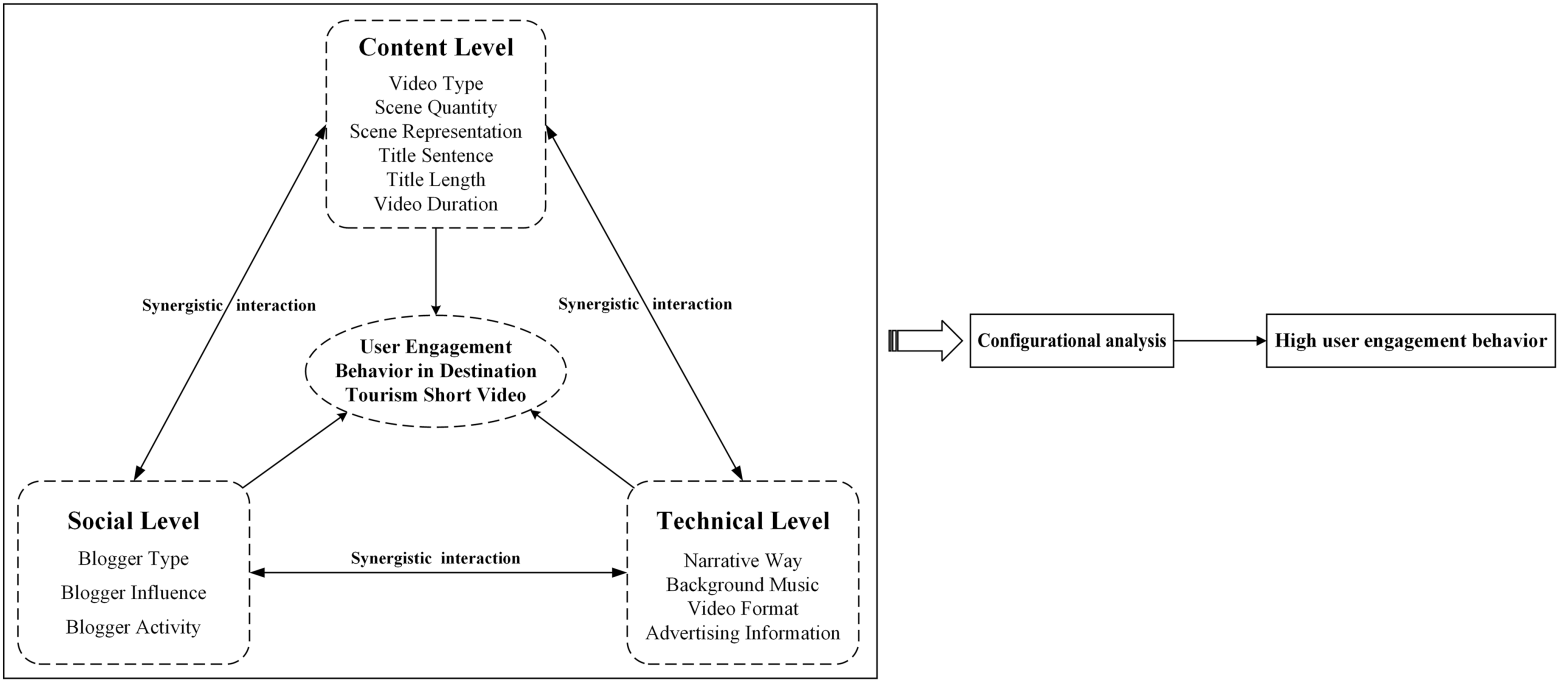
Research framework for the influencing factors of user engagement in destination tourism short video.

In summary, based on the communication ecology theory, combined with current research and the characteristics of short video components, this paper considers the influencing factors of user engagement behavior from three levels: content, social, and technical level of destination tourism short videos. It selects 13 indicators across three dimensions related to influencing factors of user engagement, which include video type, scene quantity, scene representation, title sentence, title length, video duration, blogger type, blogger influence, blogger activity, narrative way, background music, video format, and advertising information. Fully considering the complex and multifactorial nature of user engagement behavior in destination tourism short videos, this paper adopts a mixed-methods approach. Regression analysis primarily focuses on identifying key independent variables and their independent effects, providing a basis for selecting conditional variables and forming preliminary hypotheses for fsQCA. Meanwhile, fsQCA complements regression analysis by adopting a set-theoretic perspective, emphasizing the sufficiency of causal configurations and revealing complex interactions among variables. Therefore, after identifying the key factors influencing user engagement behavior, it performs configurational analysis to explore the antecedent condition configurations and pathway types leading to high user engagement behavior. The aim is to clarify the impact mechanisms of various factors and their different combinations on user engagement behavior in destination tourism short videos. Ultimately, the research framework of this study is constructed as illustrated in Fig 1.

### Data sources and acquisition

As a leading short video platform in China, TikTok has surpassed 800 million daily active users. The TikTok data platform offers multi-dimensional short video data information, which can provide robust data support for research. Therefore, TikTok (https://www.douyin.com) is selected as the data acquisition platform for this paper. Given the characteristics of short video content, which includes a vast amount of data and rapid updates, this paper uses “Xi’an Tourism” as the key theme to search for tourism short videos on TikTok. A data mining model was constructed using Python to scrape data on Xi’an tourism short videos from June 1, 2023, to June 30, 2023. A total of 5841 original short video data entries were collected, with metadata information including the visual content of the Xi’an tourism short videos, basic video information (such as titles, posting times, video durations), creator-related information (such as creator names, creator verification status, creator IDs, number of followers, number of works posted), and basic data performance of the short videos (such as the number of likes, comments, favorites, shares). Videos that were unavailable, in non-video formats, lacked updated data, or were irrelevant to the theme were cleaned out, resulting in 1,997 valid short video data entries related to Xi’an tourism, with a total video duration of approximately 1,641 minutes.

### Variable design

The dependent variable in this study is user engagement behavior of destination tourism short videos, measured by user engagement level, which is evaluated using four indicators: likes, comments, collects, and shares. Among the independent variables, variables such as title length, video duration, blogger influence, blogger activity and video format can be directly obtained from the data platform. The video type variable is extracted using the LDA topic model on the collected short video title texts. The scene quantity and scene representations are processed using computer vision technology on the obtained by the short video content. Other variables such as title sentence, blogger type, narrative way, background music, and advertising information are classified and coded manually based on existing research and the unique characteristics of short videos. The specific variable measurements are as follows.

### Quantification of dependent variable

User engagement of destination tourism short videos is conceptualized as a series of interactive actions taken by users towards short video content on social media platforms, specifically manifested through likes, comments, collects, and shares. User engagement is represented as a comprehensive quantitative expression of the degree of various engagement behaviors [23], reflecting users’ interest and approval of tourism short videos. This paper measures user engagement behavior of tourism short videos based on quantifiable data such as likes, comments, collects, and shares for each video. The formula is referred from existing research [24]:


$$W_{i}=b_{1}\ln\left(X_{1}+1\right)+b_{2}\ln\left(X_{2}+1\right)+...+b_{n}\ln\left(X_{n}+1\right)\mathrm{\backslash ]}$$ (1)

where X_1_, X_2_...X_n_ represent the number of likes, comments, collects, shares, and b_1_, b_2_...b_n_ are the weights corresponding to the indicators calculated using the entropy method.

This paper assigns weights to the number of likes, comments, collects, and shares using the entropy method. Values of 0 in these four indicators are taken as 0.000001, and natural logarithms are taken for standardization. The calculated weights for the number of likes, comments, collects, and shares are 0.2, 0.24, 0.27, and 0.29. The final expression for user engagement in destination tourism short videos is:


$$W_{i}=0.2\ln\left(X_{1}+1\right)+0.24\ln\left(X_{2}+1\right)+0.27\ln\left(X_{3}+1\right)+0.29\ln\left(X_{4}+1\right)\mathrm{\backslash ]}$$ (2)

where W_i_ represents the user engagement for the i-th tourism short video, X_1_ represents the number of likes, X_2_ represents the number of comments, X_3_ represents the number of collects, and X_4_ represents the number of shares.

### Quantification of independent variables

(1) Content level

Video type: The titles of tourism short videos exhibit the content type of the videos, and their themes can provide insights into users’ demands and preferences for short videos, serving as a key to attract viewers and engagement. The LDA topic model is used to analyze the text content of the collected short videos title texts to determine the thematic types of Xi’an tourism short videos. To ensure the effectiveness of topic clustering, the optimal number of topics is determined based on the perplexity index. Calculations show that the best clustering result is achieved when the number of topics is five. Ultimately, Xi’an tourism short videos are clustered into five thematic types: travel guides, travel recommendations, travel records, popular science information, and scenic montages.

Scene quantity: Since short videos are composed of one or more interrelated scenes, each scene is presented through a series of continuous image frames. Therefore, the core of video content analysis ultimately comes down to the analysis of individual images [25]. Using the PySceneDetect tool, we processed the collected Xi’an tourism short videos to identify the boundaries of transitions and shifts between scenes for segmentation. The central frame of each video segment is extracted as the key frame, and the scene images are saved to obtain the number of scenes contained in each tourism short video.

Scene representation: The “general object and scene recognition” function of Baidu AI open platform is invoked to identify the primary scene representation content of the key frame images of Xi’an tourism short videos. Initially, ten dimensions representing the scenes of tourism short videos are obtained, including architecture, natural landscapes, animals, and plants. Referring to existing research, modifications are made to these initial ten dimensions. Traditional architecture, modern architecture, public facilities, and transportation facilities are classified as architectural facilities. Activities involving people, such as artistic, sports, and dining entertainment, are classified as human activities. Food, clothing, crafts, and other items in the commodity category, as well as shops and malls in the architectural category, are classified as travel shopping. Animals and plants are merged into the animal and plant category. After adjustment, the scene representation variables are finally divided into five categories: architectural facilities, human activities, travel shopping, natural landscapes, and animals and plants.

Title sentence: Short videos with titles that aim to state facts and end with a period are labeled as “declarative sentences”. Titles intended to emphasize the tone and end with an exclamation mark are labeled as “exclamatory sentences”. Titles designed to elicit user discussion and end with a question mark are labeled as “interrogative sentences”. Titles containing a call to interact or hinting at interaction are labeled as “imperative sentences”. Those without titles are labeled as “no title”.

Title length: On the TikTok platform, if the short video title exceeds the display limit, the system automatically collapses part of the content, requiring users to click “expand” to view the full content. Therefore, title length variables are classified as “expand” or “not expand” based on the display mechanism of the TikTok platform.

Video duration: The video duration variable is directly quantified using the number of seconds of the tourism short video.

(2) Social level

Blogger type: The blogger type variable is measured according to the certification status of short video creators. Short videos posted by accounts certified by the government or official organizations are labeled as OGC. Those certified by professional MCN agencies are labeled as PGC. And those posted by accounts without any certification are labeled as UGC.

Blogger influence: The number of a blogger’s followers represents their influence. When creators post short video, the platform pushes the content to their followers. A larger follower count typically leads to increased views of the video. Therefore, the number of followers is used to quantify the blogger’s influence.

Blogger activity: The activity level of a blogger is measured by the frequency of short videos they publish. Bloggers who frequently post content are considered more active.

(3) Technical level

Narrative way: The narration method of short videos directly affects viewers’ understanding and perception of the content. Short videos with live human commentary are labeled as “live narration”. Those with both voiceover and subtitles are labeled as “voiceover+subtitles”. Videos with only voiceover are labeled as “voiceover”. Those with only subtitles and no audio are labeled as “subtitles”. And those without any narration are labeled as “no narration”.

Background music: Background music is also a significant technical element in short videos. Short videos with songs are labeled as “song”, music without lyrics are labeled as “absolute music”, and those without any background music are labeled as “no music”.

Video format: The video format variable is divided into “horizontal screen” and “vertical screen” according to the screen format of the short video.

Advertising information: Some creators post short videos with group-buying information attached. Based on whether group-buying information is included, this variable is divided into “yes” and “no”.

### Data analysis and research findings

#### Basic characteristics of Xi’an tourism short videos.

From Tables 1 and 2 can be seen that: In the content level, the largest category of short videos is tourism recommendations, accounting for 30.15% of the total. The highest number of scenes in tourism short video is 196, while the lowest is just one scene, with an average of approximately 19 scenes per video. Among the scenes depicted in Xi’an tourism short videos, 40.96% feature scene content related to architectural facilities, followed by scenes of human activities, which exceed 20% of the total. Scenes featuring animals and plants are relatively scarce in Xi’an tourism short videos. Most tourism short videos utilize various types of title sentences, with only 6.16% of short videos not using any sentence structure. Additionally, 89.38% of tourism short video titles are relatively short and do not require elaboration. The longest tourism short video is 1333 seconds, while the shortest is only 3 seconds, with an average duration of about 49 seconds.

**Table 1:** Continuous variable features.

Variable name	Maximum	Minimum	Mean value	Standard deviation
Scene quantity	196	1	18.790	17.638
Video duration	1333	3	49.311	71.538
Blogger influence	11264658	45	147114.703	407562.751
Blogger activity	27300	4	1172.909	2320.276
User engagement	11.077	0	3.291	2.043

**Table 2:** Categorical variable features.

Variable name	Variable type	Number of video (Strip)	Percentage (%)
Video type	Travel guides	298	14.92%
	Travel recommendations	602	30.15%
	Travel records	193	9.66%
	Popular science information	502	25.14%
	Scenic montages	402	20.13%
Scene representation	Architectural facilities	818	40.96%
	Human activities	489	24.49%
	Travel shopping	327	16.37%
	Natural landscapes	263	13.17%
	Animals and plants	100	5.01%
Title sentence	Declarative sentences	517	25.89%
	Exclamatory sentences	685	34.30%
	Interrogative sentences	195	9.76%
	Imperative sentences	477	23.89%
	No title	123	6.16%
Title length	Expand	212	10.62%
	Not expand	1785	89.38%
Blogger type	OGC	285	14.27%
	PGC	130	6.51%
	UGC	1582	79.22%
Narrative way	Live narration	507	25.39%
	Voiceover+subtitles	789	39.51%
	Voiceover	48	2.40%
	Subtitles	275	13.77%
	No narration	378	18.93%
Background music	Song	918	45.97%
	Absolute music	1007	50.43%
	No music	72	3.6%
Video format	Horizontal screen	702	35.15%
	Vertical screen	1295	64.85%
Advertising information	Yes	967	48.42%
	No	1030	51.58%

At the social level, the number of tourism short videos posted by UGC far exceeds that of OGC and PGC, accounting for 79.22% of the total. The most influential video blogger has over 11.26 million followers, whereas the least has only 45 followers, with an average follower count of approximately 147000. The most active video blogger has posted 27300 short videos, while the least has only posted 4, with bloggers averaging about 1773 short videos published.

At the technical level, only 18.93% of tourism short videos do not use any narration, while the remainder utilize various narrative ways. Most tourism short videos added instrumental music or songs as background music, with only 3.6% containing only the original sound without any background music. Vertical screen format is the most commonly used video format for tourism short videos. Additionally, 48.42% of tourism short videos are published alongside advertising information. The maximum user engagement for Xi’an tourism short videos is 11.077, the minimum is 0, and the average is 3.291.

### Regression analysis of factors influencing user engagement in Xi’an tourism short videos

A multiple regression analysis was conducted on the influencing factors at various levels, including content, social, and technical levels, to examine the causal relationships between each independent variable and user engagement behavior in Xi’an tourism short videos. This provides a basis for calibrating the data analysis in the subsequent fsQCA analysis. Since the independent variables comprised both continuous and categorical variables, categorical variables were converted into dummy variables for convenient calculation before regression analysis. In the regression model of factors influencing Xi’an tourism short videos, the VIF values of all independent variables were less than five, indicating no multicollinearity issues. The model demonstrated good fit, allowing all independent variables to be included in the regression model for calculation, with the regression results presented in Table 3.

**Table 3:** Regression analysis results of influencing factors for user engagement of tourism short videos in Xi’an.

Level	Variable name	Variable type	User engagement	
			Beta	VIF
Content level	Video type	(Take the travel guides as a reference)		
		Travel recommendations	−0.120***	2.401
		Travel records	−0.100***	1.602
		Popular science information	−0.094**	2.270
		Scenic montages	−0.092**	2.660
	Scene quantity	/	0.173***	2.193
	Scene representation	(Take the natural landscapes as a reference)		
		Architectural facilities	−0.085**	2.737
		Human activities	−0.088**	2.683
		Travel shopping	−0.136***	2.305
		Animals and plants	−0.051*	1.398
	Title sentence	(Take the no title as a reference)		
		Declarative sentences	0.094*	4.047
		Exclamatory sentences	0.225***	4.586
		Interrogative sentences	0.123***	2.436
		Imperative sentences	0.133***	4.076
	Title length	Expand or not expand	−0.018	1.061
	Video duration	/	0.022	1.983
Social level	Blogger type	(Take the OGC as a reference)		
		PGC	0.054*	1.510
		UGC	0.144***	1.811
	Blogger influence	/	0.281***	1.156
	Blogger activity	/	−0.180***	1.211
Technical level	Narrative way	(Take the live narration as a reference)		
		Voiceover+Subtitles	−0.212***	1.742
		Voiceover	−0.081***	1.136
		Subtitles	−0.246***	1.699
		No narration	−0.128***	2.448
	Background music	(Take the absolute music as a reference)		
		Song	0.054**	1.101
		No music	0.052**	1.147
	Video format	Horizontal screen or vertical screen	−0.020	1.142
	Advertising information	Yes or no	−0.100***	1.471
R^2^			0.324	

Note: *p < 0.05 **p < 0.01 ***p < 0.001.

At the content level, compared to tourism short videos featuring travel guides, the user engagement in other types of tourism short videos such as travel recommendations(B = −0.120, p < 0.001), travel records(B = −0.100, p < 0.001), popular science information(B = −0.094, p < 0.01), and scenic montages(B = −0.092, p < 0.01) showed significant negative correlations. This is because travel guide videos better meet users’ needs for practical information about destinations during their visit to Xi’an. The number of scenes shown in tourism short videos positively impacts user engagement(B = 0.173, p < 0.001). When more Xi’an travel scenes are captured, users are often attracted by their rich visual content and actively engage and interact. Conversely, a relatively monotonous visual scene would diminish user interest and reduce their engagement. Compared to short videos featuring natural landscapes, videos primarily depicting architectural facilities (B = −0.085, p < 0.01), human activities (B = −0.088, p < 0.01), tourism shopping (B = −0.136, p < 0.001), animals and plants (B = −0.051, p < 0.05) negatively affect user engagement. Tourism short videos with titles using declarative sentences (B = 0.094, p < 0.05), exclamation sentences (B = 0.225, p < 0.001), interrogative sentences (B = 0.123, p < 0.001), or imperative sentences (B = 0.133, p < 0.001) attract users’ attention and increase their willingness to engage more effectively than videos with no title. Additionally, the title length (B = −0.018, p > 0.05) and video duration (B = 0.022, p > 0.05) of tourism short videos did not significantly impact user engagement, suggesting that users are more focused on the quality of the content itself.

Social level factors all have significant impacts on user engagement. For instance, compared to tourism short videos published by OGC, those published by UGC (B = 0.144, p < 0.001) or PGC (B = 0.054, p < 0.05) positively influence user engagement. This is because content published by UGC or PGC is closer to users’ needs, thus easily gaining users’ trust and resonance. The influence of bloggers significantly positively affects user engagement (B = 0.281, p < 0.001), indicating that the larger blogger’s fanbase, the greater their influence and ability to mobilize users to engage with the tourism short videos they publish. Moreover, the blogger’s activity level shows a significant negative correlation with user engagement (B = −0.180, p < 0.001), suggesting that if bloggers post tourism short videos too frequently, it may lower user engagement. This is because excessive content potentially distracts users’ attention, causing aesthetic fatigue and reducing interest in short videos.

At the technical level, compared to tourism short videos with live narration, videos utilizing voiceover+subtitles (B = −0.212, p < 0.001), voiceover only (B = −0.081, p < 0.001), subtitles only (B = −0.246, p < 0.001), or no narration (B = −0.128, p < 0.001) negatively impact user engagement. This is because short videos with live narration are generally more vivid and cordial, bringing them closer to the audience. Regarding background music selection, using songs (B = 0.054, p < 0.01) or no music (B = 0.052, p < 0.01) positively influences user engagement compared to using absolute music. There is no significant difference in the effect on user engagement between vertical and horizontal tourism short video formats (B = −0.020, p > 0.05), indicating that users are not sensitive to the browsing format of short videos. Furthermore, advertising information has a significant negative impact on user engagement (B = −0.100, p < 0.001), suggesting that excessive advertising information can interfere with users’ viewing experience, reducing their interest and satisfaction with the content.

### Configurational analysis of factors influencing user engagement in Xi’an tourism short videos

#### Data calibration.

Due to the insignificant impact of the independent variables such as title length, video duration, and video format on user engagement behavior, these variables are excluded from the configurational analysis. Based on the results of the regression analysis, the remaining variables are calibrated to transform the data into a set concept between 0 and 1. Drawing from existing research [26], we employ the direct calibration method for continuous independent variables and user engagement, selecting the 95%, 50%, and 5% quantiles of the data as anchors for complete membership, crossover points, and complete non-membership. Dichotomous variables are assigned values of 0 and 1 without requiring calibration, while the remaining categorical variables are calibrated using the indirect calibration method. The absolute value of the regression coefficient to some extent reflects the degree of influence of a variable on user engagement, with variables having larger regression coefficients exerting a greater impact on the outcome. Consequently, regression coefficients were utilized to assist in adjusting the quantile anchor points. For categorical variables with larger regression coefficients, a higher membership value was assigned during the calibration process, making them more likely to belong to that variable. Conversely, variables with smaller regression coefficients were assigned a relatively lower membership value. To ensure the completeness of the cases, any variables calibrated to 0.5 are incremented by 0.001 [27]. The calibration results are presented in Tables 4 and 5.

**Table 4:** Calibration of continuous variable data.

Variable type	Variable name	Anchor point		
		Complete membership (Membership 95%)	Crossover point (Membership 50%)	Complete non-membership (Membership 5%)
Conditional variable	Scene quantity	51	14	2
	Video duration	160	31	9
	Blogger influence	622750	38036	9964
	Blogger activity	64	577	3700
Outcome variable	User engagement	6.832218542	2.973981802	0.678554735

**Table 5:** Calibration of categorical variable data.

Variable name	Variable type	Assignment
Video type	Travel guides	1
	Travel recommendations	0.75
	Travel records	0.5
	Popular science information	0.25
	Scenic montages	0
Scene representation	Natural landscapes	1
	Travel shopping	0.75
	Human activities	0.5
	Architectural facilities	0.25
	Animals and plants	0
Title sentence	Exclamatory sentences	1
	Imperative sentences	0.75
	Interrogative sentences	0.5
	Declarative sentences	0.25
	No title	0
Blogger type	UGC	1
	PGC	0.5
	OGC	0
Narrative way	Live narration	1
	Subtitles	0.75
	Voiceover+Subtitles	0.5
	No narration	0.25
	Voiceover	0
Background music	Song	1
	No music	0.5
	Absolute music	0

### Necessary condition analysis

Before conducting configurational analysis, it is necessary to perform a necessity test on individual conditional variables to determine if there are any necessary conditions that lead to the occurrence of the outcome variable. A variable can be considered a necessary condition for the outcome variable when its consistency level exceeds 0.9 [26]. The calibrated data is imported into fsQCA software for necessity analysis, and the results are presented in Table 6. No condition variables records a consistency level greater than 0.9, indicating that there are no necessary factors that solely influence high user engagement in tourism short videos. No single factor can independently determine user engagement behavior in tourism short videos. Therefore, a configurational analysis of the antecedent variables influencing user engagement behavior is necessary to explore the combinational driving effects among the various factors.

**Table 6:** Necessity analysis of single conditions.

Conditional variable		User engagement	
		Consistency	Coverage
Content level	Video type	0.628508	0.614422
	~Video type	0.599419	0.555342
	Scene quantity	0.675000	0.704654
	~Scene quantity	0.615496	0.537844
	Scene representation	0.623939	0.618845
	~Scene representation	0.666726	0.609404
	Title sentence	0.780840	0.584182
	~Title sentence	0.430667	0.562483
Social level	Blogger type	0.858423	0.495061
	~Blogger type	0.168904	0.458584
	Blogger influence	0.665651	0.781955
	~Blogger influence	0.638513	0.510391
	Blogger activity	0.774358	0.654480
	~Blogger activity	0.514830	0.560129
Technical level	Narrative way	0.768045	0.606450
	~Narrative way	0.507395	0.607058
	Background music	0.494921	0.492765
	~Background music	0.520177	0.473785
	Advertising information	0.440071	0.432296
	~Advertising information	0.559929	0.516394

### Conditional configuration analysis

To further explore the combined effects of conditional variables, a truth table was constructed in the fsQCA software for configuration analysis. Following the operational recommendations from existing research [27], the consistency threshold was set at 0.8, the frequency threshold at 10, and the PRI threshold at 0.7, resulting in complex solutions, parsimonious solutions, and intermediate solutions. The results showed that the complex solution was consistent with the intermediate solution in this case, with the intermediate solution generating six pathways and the parsimonious solution generating four pathways. By combining the intermediate and parsimonious solutions, core and peripheral conditions influencing high user engagement behavior were identified. Factors that appeared only in the intermediate solution were considered as peripheral conditions, while those present in both the parsimonious and intermediate solutions were considered core conditions. Ultimately, six configuration results that drive high user engagement in tourism short videos were formed, as shown in Table 7. The consistency level of the individual configuration solutions were above 0.8, and the overall consistency level of 0.907653, indicating that these six configurations have a strong explanatory power for the selected cases. The coverage of the overall solution was 0.30185, indicating that these six configurations can explain 30.19% of the short video cases. This is comparable to the research using QCA methods in the fields of organization and management, and meets the standards of fsQCA [28–31]. To demonstrate the robustness of these configuration results, the consistency threshold was adjusted to lower levels of 0.75 and higher levels of 0.85 for comparison following the practices of related research [32]. The resulting configuration outcomes remained consistent with previous findings, proving the reliability of this study’s results.

**Table 7:** Configuration analysis of antecedent conditions.

Conditional variable	Configuration path					
	1	2	3	4	5	6
Video type	●	●	●	●	●	
Scene quantity	●	●	●	●	●	●
Scene representation		●	⊗	⊗	⊗	⊗
Title sentence	●	●	●	●		●
Blogger type	●	●	●	●	●	●
Blogger influence	●	●	●		●	●
Blogger activity	●	●		●	●	●
Narrative way	●	●	●	●	●	●
Background music			⊗	⊗	⊗	⊗
Advertising information	⊗		⊗	⊗	⊗	⊗
Raw coverage	0.128105	0.252746	0.0665085	0.0731585	0.0714878	0.077534
Unique coverage	0.00818679	0.145506	0.00139636	0.00804621	0.00637558	0.0124217
Consistency	0.945394	0.917914	0.932508	0.894176	0.936127	0.943618
Overall solution coverage	0.30185					
Overall solution consistency	0.907653					

Note: ●core conditions exist ⊗core conditions absence ●edge conditions exist ⊗edge conditions absence, blanks indicate that the condition variable is irrelevant to the outcome.

Configuration 1 (VT*SQ*TS*BT*BI*BA*NW* ~ AI): Configuration 1 shows that video type, scene quantity, blogger type, blogger influence, blogger activity, and non-advertising information serve as core conditions, while title sentence and narrative way as peripheral conditions, capable of generating higher user engagement. This suggests that tourism short videos focusing on travel guides, featuring rich visual scenes, and employing eye-catching exclamatory titles are likely to capture user attention. If these videos utilize engaging narrative ways, such as personal appearances, while simultaneously reducing the frequency of tourism advertisement to maintain the purity of the viewing experience, they can achieve active user engagement and interaction based on their strong credibility and large fan base, even if the content creator publishes a limited amount of short videos.

Configuration 2 (VT*SQ*SR*TS*BT*BI*BA*NW): Configuration 2 shows that video type, scene quantity, scene representation, blogger influence, and blogger activity are core conditions, while title sentence, blogger type, and narrative way are peripheral conditions that can easily elicit high user engagement. This implies that if tourism short videos possess characteristics that meet user preferences, such as popular travel guides, rich visual scenes, natural landscapes content that aligns with user needs, and engaging exclamatory titles, even if the blogger is not very active in posting videos, as long as they have high authenticity and credibility with a large following, and the narrative way is engaging and well-suited to the content, it can stimulate user engagement and achieve good interaction effects.

Configuration 3 (VT*SQ* ~ SR*TS*BT*BI*NW* ~ BM* ~ AI): Configuration 3 reveals that video type, scene quantity, blogger type, blogger influence, and non-advertising information are core conditions, while non-scene representation, title sentence, narrative way, and non-background music are peripheral conditions capable of triggering high user engagement. This indicates that for scenes lacking user attention, such as featuring animals and plants, and for tourism short videos posted infrequently by bloggers, other means are needed to capture users’ attention. User-preferred travel guide videos, rich visual scenes, exciting exclamatory titles, background music that is soothing absolute music, as well as the blogger’s credibility and fan base, are all crucial for enhancing user engagement. Additionally, by understanding user needs and incorporating vivid personal appearances in the narrative way, while avoiding excessive advertisement interruptions, user engagement can be actively achieved.

Configuration 4 (VT*SQ* ~ SR*TS*BT*BA*NW* ~ BM* ~ AI): Configuration 4 indicates that the blogger activity and non-advertising content serve as the core conditions, while video type, scene quantity, non-scene representation, title sentence, blogger type, narrative way, and non-background music serve as peripheral conditions that can lead to high user engagement. This implies that even if the posting rate of tourism short videos is low and the represented content depicts less user-desirable scenes like plants and animals, high user engagement can still be achieved by focusing on video types that cater to users’ preferences for travel guides, enriching the visual scenes, using exclamatory titles that align with user interests, and ensuring that the blogger is trusted by users. Additionally, ensuring that the narrative way and background music match audience preferences while minimizing unnecessary advertising information can still garner user affection and active engagement.

Configuration 5 (VT*SQ* ~ SR*BT*BI*BA*NW* ~ BM* ~ AI): Configuration 5 shows that video type, scene quantity, blogger type, blogger influence, blogger activity, and non-advertising content serve as the core conditions, while non-scene representation, narrative way, and non-background music serve as peripheral conditions that can generate high user engagement. This suggests that in the case of tourism short videos characterized by user-favored travel guides, rich visual scenes and absolute background music, if the blogger can win user trust and has a large fan base, they can still yield favorable user engagement and feedback by using genuine personal narration and minimizing the push of advertising information, even if the posting rate is low and the represented content includes animals and plants that deviate somewhat from user needs.

Configuration 6 (SQ* ~ SR*TS*BT*BI*BA*NW* ~ BM* ~ AI): Configuration 6 reveals that scene quantity, blogger type, blogger influence, blogger activity, and non-advertising information serve as the core conditions, while non-scene representation, title sentence, narrative way, and non-background music serve as peripheral conditions that can promote high user engagement. This means that when tourism short videos represent content such as animals and plants that are not to users’ liking, and the blogger is trusted by users, with a good fan base but posts infrequently, the key to attracting users’ attention and achieving good user engagement lies in ensuring the richness of video scenes, crafting eye-catching exclamatory titles, selecting background music that caters to users’ preferences, minimizing the placement of advertising information that may irritate users.

### Interpretation of configuration paths

**Fig 2:**
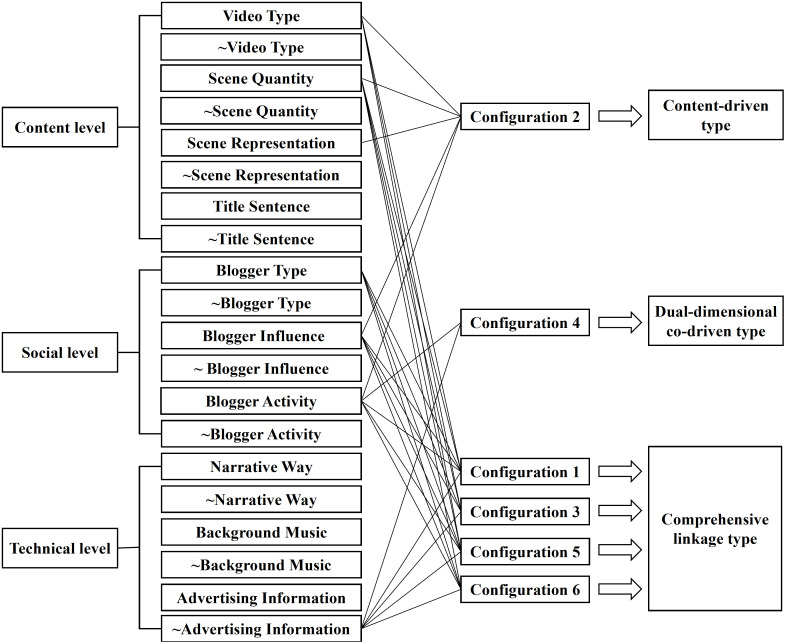
Influence path of user engagement behavior in destination tourism short video.

Fully consider the intrinsic connections between various factors, and further refine and summarize based on the distribution characteristics of core conditions in each condition configuration, thus outlining the path patterns that lead to high user engagement behavior in destination tourism short videos. A comparative analysis of each antecedent condition configuration reveals that social factors are present in all six configurations, indicating that social factors serve as a significant driving force for high user engagement in tourism short videos. In Configuration 2, core conditions are found only in content and social levels, lacking core factors from the technical level. Based on the connotations of the influencing factors involved in this configuration, this path is defined as content-driven type. In Configuration 4, core conditions appear only in technical and social levels, with an absence of core elements from the content level. Therefore, this path is defined as dual-dimensional co-driven type. Configurations 1, 3, 5, and 6 share the commonality of comprehensive antecedent conditions, with core conditions emerging in content, social, and technical levels. By synthesizing the connotations of the influencing factors across these levels, this type of configuration path is defined as integrated and interconnected. Ultimately, three types of configuration path that trigger user engagement in destination tourism short videos are identified: content-driven, dual-dimensional co-driven, and comprehensive linkage, allowing destination marketers to select appropriate models for precise marketing of tourism short videos based on their development situations. The influence path is shown in Fig 2.

Content-driven type: This path type primarily includes Configuration 2, where core conditions consist of elements from the content and social levels that prompt users to exhibit high engagement in tourism short videos. These tourism short video strives to create compelling and attractive content, increasing follower counts through high-quality content output and forming a stable audience for short videos. This suggests that high-quality video content and effective fan operation are important combined factors that trigger user engagement in tourism short videos. The key to the content-driven type lies in creators needing to clarify the message theme they wish to convey and produce original content infused with cultural and emotional elements or embodying a distinctive destination image. By comprehensively showcasing the charm of the destination through multiple perspectives and utilizing shifts in different perspectives, users can feel an immersive experience. Creators should skillfully utilize combinations of different shot scales to increase the layered sense of the image and reduce visual fatigue for users. By setting captivating titles to attract users’ attention, and stimulate users’ interest in participating in short videos. This short video model with high-quality content as its core excels in stimulating user engagement and interaction, enhancing audiences’ awareness and memory of tourist destinations.

Dual-dimensional co-driven type: This path type primarily encompasses Configuration 4, which revolves around the technical and social levels to foster high user engagement in short tourism videos. These videos strive for seamless visual and auditory integration, capturing public attention and enhancing user retention rates through unique creative styles. This demonstrates that interesting presentation formats and effective fan management are crucial in attracting user engagement in tourism short videos. The focus of the dual-dimensional co-driven type lies in adopting suitable narrative ways to help users better understand the video content, enhance the video’s authenticity and approachability through real people appearing on screen, and enhance users’ browsing experience through strong visual and auditory impact. Appropriate music is selected based on the content and theme of the short videos to intensify the atmosphere and emotional expression of the scenes, while various audio effects are added in specific scenes or activities to create a sense of realism and immersion. Creative methods are utilized to insert short video advertisements, maintaining a natural and smooth content transition, reducing the sense of incongruity of advertisements, and minimizing users’ aversion to tourism advertisements.

Comprehensive linkage type: This path primarily corresponds to configurations 1, 3, 5, and 6, emphasizing the balanced role of content, social, and technical levels in jointly promoting users’ engagement in tourism short videos. These short videos have certain requirements for various attributes, emphasizing not only rich and fulfilling content but also skilled use of technological means to evoke emotions, while also paying attention to cultivating a loyal fan base. It comprehensively expresses and interprets short videos in a holistic manner, prompting users to engage in interactions more actively. The comprehensive linkage type should focus on deeply understanding the needs and psychology of the audience, analyzing users’ pain points, and accurately pushing destination information to users. It is essential to ensure a creative and practical theme for entry, integrating local elements with current hot topics to increase the exposure of short videos. Attention should be paid to the use of visual and scenic elements, showcasing the unique characteristics of tourist destinations from different perspectives. Engaging narration, appropriate background music, and special effects should be added to attract users’ attention, avoiding monotony or clutter to enhance the video’s enjoyment and emotional impact. Creators can collaborate with internet celebrities and travel experts, leveraging their influence and fanbase to expand the reach of short videos among users. When presenting short video content, initiatives such as challenges, polls, and lotteries can be launched to guide tourists to participate in interactions, prompting users to convert their interests into actual tourism activities.

## Conclusion and discussion

### Conclusion

Tourism short videos provide a platform for destinations to showcase their charm. These meticulously produced tourism short videos not only offer valuable tourism information and enhance viewers’ awareness and understanding of the destination, but also spark potential tourists’ interest and encourage active engagement, ultimately generating a desire to visit personally. Taking Xi’an, a popular city on short video platforms, as an example, this paper constructs a research framework for the influencing factors of user engagement behavior in tourism short videos based on communication ecology theory from the content, social, and technical levels. By combining LDA topic analysis and computer vision technology to obtain crucial data required for the research, it provides significant support for an in-depth exploration of the factors influencing user engagement in tourism short videos. Meanwhile, the study fully considers the complex mechanisms of user engagement from a configurational perspective, employing a mixed method of multiple regression and fsQCA to explore the key factors and configurational paths that influence user engagement in destination tourism short videos.

The research findings reveal that factors such as video type, scene quantity, scene representation, and title sentence at the content level significantly impact user engagement behavior in destination tourism short videos. At the social level, blogger type, blogger influence, and blogger activity all have notable effects on user engagement behavior. Additionally, factors such as narrative way, background music, and advertising information at the technical level significantly influence user engagement behavior. The necessity analysis shows that no single factor can independently affect user engagement, and elements across different dimensions exert an interactive and combined effect on user engagement behavior. However, the social level factors demonstrate a significant and universal role in promoting high user engagement with tourism short videos. which is evidenced by the effective promotion of user engagement through maintaining a good fan base, increasing influencer popularity, and maintaining appropriate activity levels. Multiple factors interact to influence users’ engagement behaviors in tourism short videos. The fsQCA method identifies six conditional configurations that trigger active user engagement. Based on core conditions, the paths leading to high user engagement are classified into three logical types: content-driven, dual-dimensional co-driven, and comprehensive linkage. In the content production and promotion of tourism short videos, high-quality content is the core in stimulating user interest, technical support is crucial, and effective fan management plays an important facilitating role.

## Discussion

### Theoretical contributions

Short videos are playing an increasingly significant role in promoting the development of the tourism industry and attracting tourists to explore destinations, becoming an indispensable part of tourism marketing. However, compared with research on tourism-related images and texts, there are relatively few studies focusing specifically on tourism short videos, especially the exploration of user engagement behavior in destination tourism short videos. This study delves into the driving mechanisms and complex causal relationships behind users’ active engagement in tourism short videos, thereby enriching the research content in this field. Secondly, this study differs from existing research that employs traditional methods to explore the factors influencing user behavior. By integrating computer vision technology into the context of tourism short video research to uncover the factors affecting user engagement, thereby broadening the application of interdisciplinary theories and techniques in tourism short video research. Furthermore, existing research tends to investigate the mechanisms of single or multiple influencing factors in isolation, lacking a comprehensive integration of multifaceted factors. Based on communication ecology theory, this paper constructs a research framework for the influencing factors of user engagement behavior in tourism short videos. Using a mixed-methods, this study analyzes the intrinsic patterns of user engagement. From a configurational perspective, it reveals the complex causal relationships resulting from the simultaneous involvement of multiple factors in user engagement. This provides new ideas for the study of user engagement behavior in destination tourism short videos. Additionally, this study identifies diverse pathways that trigger high user engagement with tourism short videos and categorizes three types of marketing pathways that stimulate user engagement. It can help destination tourism marketers understand user needs, adjust and optimize tourism marketing strategies based on tourists’ behavioral characteristics and demand preferences, thereby enabling destination to achieve effective tourism short video promotion and marketing results.

### Managerial implications

The findings of this study have important reference value for understanding the intrinsic laws of user engagement behavior and achieving precise marketing for tourism destinations. By gaining insights into users’ expectations and behavioral preferences for engaging in tourism short videos, destination managers can adopt corresponding strategies for quality improvement and upgrading. Social-level factors are reflected across all six configurations, indicating their consistent role in generating high user engagement in tourism short videos. Content creators effectively promote the destination’s image and tourism product marketing. Other core conditions mainly stem from content-level factors, where interesting and rich video content plays a crucial role. In contrast, technological-level factors primarily serve as marginal conditions, providing auxiliary support. Three types of pathways can stimulate users’ active engagement in destination tourism short videos. Destination marketers should choose an appropriate model for precise marketing of tourism short videos based on their development circumstances.

Tourists who prefer the content-driven configurational path model have higher requirements for the content quality of tourism short videos, hoping to obtain more attractive destination travel stories or information. Therefore, destination tourism marketers should deeply understand the potential needs of users, accurately grasp the core pain points of tourists, and reshape the tourist value of the destination by actively exploring destination characteristics. At the same time, they should strengthen content innovation in tourism short videos and create unique memory points to provide tourists with high-quality tourism marketing content. Tourists who prefer the dual-dimension co-driven configurational path model attach great importance to the visual effects of tourism short video production and creator’s user maintenance. Destination tourism marketers need to keep up with social development trends, continuously optimize tourism short video production techniques, and create a good scene atmosphere. While ensuring technical aesthetics, they should also focus on establishing emotional resonance with tourists, actively strengthen connections and interactions with tourists, shape the collective memory of tourist groups, and enhance user stickiness and the influence of creators, thereby promoting tourists’ active participation in destination tourism short videos. Tourists who prefer the comprehensive and interconnected configurational path model tend to pursue a comprehensive experience and value the overall presentation of tourism short videos. Whether it’s unappealing video content, lack of aesthetic appeal and viewing pleasure, or absence of fan interaction and operation, all these can quickly lead to a loss of interest among users and potential tourist attrition. Therefore, destination tourism marketers need to cover the diverse needs of tourists, integrate and coordinate elements at the content, social, and technical levels, and work together from multiple aspects to build a good tourism short video ecosystem. They should enhance the visual aesthetics, overall texture, and audience appeal of tourism short video content production and promotion, thereby providing tourists with an immersive sensory experience. Destination tourism marketers can create differentiated tourism short video content tailored to the characteristics of users with different preferences, in order to precisely reach target tourist groups and effectively attract users’ attention and participation. Through precise marketing of tourism short videos, tourists can develop a strong interest and desire for the destination while browsing and engaging with the videos. This converts online traffic into actual tourism enthusiasm, promoting sustainable and high-quality development of tourism destinations.

### Limitations and future directions

Whether users engagement in interactive tourism short videos is a complex result of multiple factors. Besides the factors discussed in this paper, there may be other unexplored factors. Future research needs to further explore these potential factors to more comprehensively reveal the internal mechanisms of user engagement in tourism short videos. Different social media platforms have somewhat different user demographics and positioning. Future studies can examine and compare user engagement behavior in destination tourism short videos on different platforms to enhance the universality of the results.

## Supporting information

S1 Data Research Data. (XLSX)
